# *ANGPT2* and *NOS3* Polymorphisms and Clinical Outcome in Advanced Hepatocellular Carcinoma Patients Receiving Sorafenib

**DOI:** 10.3390/cancers11071023

**Published:** 2019-07-20

**Authors:** Giorgia Marisi, Elisabetta Petracci, Francesco Raimondi, Luca Faloppi, Francesco Giuseppe Foschi, Gianfranco Lauletta, Massimo Iavarone, Matteo Canale, Martina Valgiusti, Luca Maria Neri, Paola Ulivi, Giulia Orsi, Giulia Rovesti, Ranka Vukotic, Fabio Conti, Alessandro Cucchetti, Giorgio Ercolani, Kalliopi Andrikou, Stefano Cascinu, Mario Scartozzi, Andrea Casadei-Gardini

**Affiliations:** 1 Biosciences Laboratory, Istituto Scientifico Romagnolo per lo Studio e la Cura dei Tumori (IRST) IRCCS, 47014 Meldola, Italy; 2 Unit of Biostatistics and Clinical Trials, Istituto Scientifico Romagnolo per lo Studio e la Cura dei Tumori (IRST) IRCCS, 47014 Meldola, Italy; 3 CellNetworks—Cluster of Excellence (EXC81) and Biochemistry Center (BZH), Heidelberg University, 69120 Heidelberg, Germany; 4 Department of Experimental Oncology, IEO, European Institute of Oncology IRCCS, 20141 Milan, Italy; 5 Department of Medical Oncology, University Hospital Cagliari, 09124 Cagliari, Italy; 6 DPT Internal Medicine, Faenza Hospital, AUSL Romagna, 48018 Faenza, Italy; 7 Department of Biomedical Sciences and Human Oncology, Internal Medicine “G. Baccelli”, University of Bari “A. Moro”, 70121 Bari, Italy; 8 “A.M.&A. Migliavacca” Center for Liver Disease, 1st Division of Gastroenterology, Fondazione IRCCS Ca’ Granda Maggiore Hospital, University of Milan, 20122 Milan, Italy; 9 Department of Medical Oncology, Istituto Scientifico Romagnolo per lo Studio e la Cura dei Tumori (IRST) IRCCS, 47014 Meldola, Italy; 10 Department of Morphology, Surgery and Experimental Medicine, University of Ferrara, 44121 Ferrara, Italy; 11 Division of Oncology, Department of Medical and Surgical Sciences for Children & Adults, University-Hospital of Modena and Reggio Emilia, 41121 Modena, Italy; 12 Department of Medical and Surgical Sciences, University of Bologna, 40138 Bologna, Italy; 13 General and Oncology Surgery, Morgagni-Pierantoni Hospital, 47121 Forli, Italy; 14 Department of Medical & Surgical Sciences—DIMEC, Alma Mater Studiorum—University of Bologna, 40138 Bologna, Italy

**Keywords:** single-nucleotide polymorphism, angiopoietin, endothelial nitric oxide synthase, hepatocellular carcinoma, biomarkers, angiogenesis, Child–Pugh, VEGF

## Abstract

Sorafenib represents the standard of care for advanced hepatocellular carcinoma (HCC), even though a large number of patients have reported limited efficacy. The aim of the present study was to evaluate the prognostic value of single-nucleotide polymorphisms on angiopoietin-2 (*ANGPT2*) and endothelial-derived nitric oxide synthase (*NOS3*) genes in 135 patients with advanced HCC receiving sorafenib. Eight *ANGPT2* polymorphisms were analyzed by direct sequencing in relation to overall survival (OS) and progression-free survival (PFS). In univariate analysis, *ANGPT2*rs55633437 and *NOS3* rs2070744 were associated with OS and PFS. In particular, patients with *ANGPT2*rs55633437 TT/GT genotypes had significantly lower median OS (4.66 vs. 15.5 months, hazard ratio (HR) 4.86, 95% CI 2.73–8.67, *p* < 0.001) and PFS (1.58 vs. 6.27 months, HR 4.79, 95% CI 2.73–8.35, *p* < 0.001) than those homozygous for the G allele. Moreover, patients with *NOS3* rs2070744 TC/CC genotypes had significantly higher median OS (15.6 vs. 9.1 months, HR 0.65, 95% CI 0.44–0.97; *p =* 0.036) and PFS (7.03 vs. 3.5 months, HR 0.43, 95% CI 0.30–0.63; *p* < 0.001) than patients homozygous for the T allele. Multivariate analysis confirmed these polymorphisms as independent prognostic factors. Our results suggest that *ANGPT2*rs55633437 and *NOS3* rs2070744 polymorphisms could identify a subset of HCC patients more resistant to sorafenib.

## 1. Introduction

Primary liver cancer represents the sixth most common cancer and the third most frequent cause of cancer-related death worldwide [1]. Hepatocellular carcinoma (HCC) is the most common type of primary liver cancer, and despite new therapeutic approaches, prognosis remains poor.

Patients with advanced-stage disease can benefit from systemic therapies. Sorafenib, a multi-target tyrosine kinase inhibitor, has been considered the standard of care for patients with advanced unresectable HCC since 2007 [2], but it is expensive and associated with adverse events (AEs). Furthermore, a proportion of patients show no response to sorafenib, and molecular predictors of its efficacy have not yet been identified [3].

Moreover, given the prominent arrival of new drugs in this setting [4], it would be useful to have biomarkers capable of identifying those patients who are more likely to benefit from certain therapies.

Angiogenesis is one of the pathways most involved in the mechanism of action of sorafenib, and numerous studies have focused on the role of markers involved in the angiogenesis process at both the expression and genetic levels [3,5]. The largest biomarker study conducted to date is the SHARP trial, in which baseline angiopoietin-2 (Ang-2) plasma levels independently predicted survival in both the entire patient population and the placebo cohort [5].

Ang-2 is an angiogenic factor that binds Tie2 receptor and cooperates with the vascular endothelial growth factor (VEGF) pathway to maintain physiological functions. Genetic variants in the Ang-2 gene (*ANGPT2*) may lead to altered activities of the gene.

In our previous retrospective study, we analyzed three endothelium-derived nitric oxide synthase (*NOS3*) polymorphisms located inside the *NOS3* gene on chromosome 7. We demonstrated that patients with the *NOS3* rs2070744 (*NOS3-786*) TT genotype had significantly shorter median progression-free survival (PFS) and overall survival (OS) when compared to those with other genotypes [6].

The primary aim of the current study was to investigate the prognostic value of different single-nucleotide polymorphisms (SNPs) on *ANGPT2* and *NOS3* genes in relation to OS in patients with advanced HCC receiving sorafenib treatment. The second aim was to verify whether these polymorphisms are related or not to progression-free survival (PFS), disease control rate (DCR), and toxicities.

## 2. Results

### 2.1. Patient Characteristics and Clinical Variables

The main clinical pathological characteristics of patients are shown in Table 1. The median follow-up for PFS was 2.96 months (95% CI: 1.87–3.91), whereas that for OS was 8.9 months (1.71–48.92). The median PFS was 5.75 months (95% CI: 5.06–6.60), and the median OS was 14.39 months (95% CI: 11.83–15.74).

Univariate analyses regarding PFS and OS data in relation to baseline patient characteristics are shown in Table 2. In particular, we found that patients with hepatitis B virus (HBV) etiology showed worse OS than patients with hepatitis C virus (HCV) etiology (8.57 vs. 14.3 months; hazard ratio (HR) 1.95, 95% CI: 1.17–3.26; *p* = 0.011), and patients without extra-hepatic spread showed better outcomes in terms of PFS (6.27 vs. 2.83 months; HR 0.50, 95% CI: 0.34–0.73; *p* < 0.001) and OS (15.6 vs. 10.84 months; HR 0.65, 95% CI: 0.43–0.99; *p* = 0.043) than patients with metastatic disease. No significant correlation was found between the other clinical characteristics and clinical outcomes.

### 2.2. ANGPT2 and NOS3 Genotypes and Clinical Outcomes

The genotype frequencies of *ANGPT2* and *NOS3* polymorphisms are shown in Table S1, and all followed the Hardy–Weinberg equilibrium. Missing data on polymorphisms are due to the lack of input DNA. Some *ANGPT2* and *NOS3* SNPs were highly correlated to each other (Table S2).

**Figure 1:**
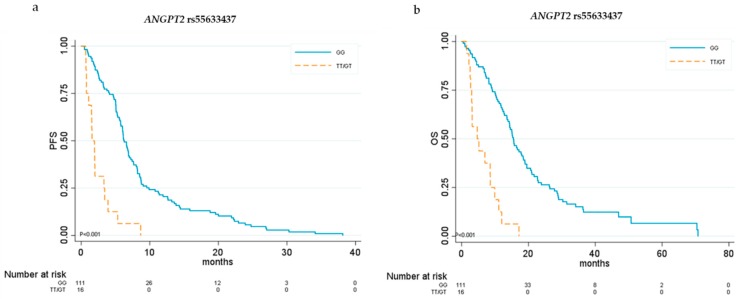
Kaplan–Meier curves for *ANGPT2* rs55633437. (**a**) Progression-free survival (PFS) and (**b**) overall survival (OS) in relation to *ANGPT2* rs55633437 genotypes.

By univariate analysis we found that three *ANGPT2* SNPs and two *NOS3* SNPs were associated with clinical outcomes. In particular, *ANGPT2* SNP rs55633437 was associated with both PFS and OS. For this polymorphism, we chose the dominant genetic model. Patients with at least one copy of the minor allele T had significantly lower median PFS (1.58 vs. 6.27 months, HR 4.79, 95% CI 2.73–8.35; *p* < 0.001) and OS (4.66 vs. 15.51 months, HR 4.86, 95% CI 2.73–8.67; *p* < 0.001) than did those homozygous for the G allele (Table 3 and Figure 1).

**Figure 2:**
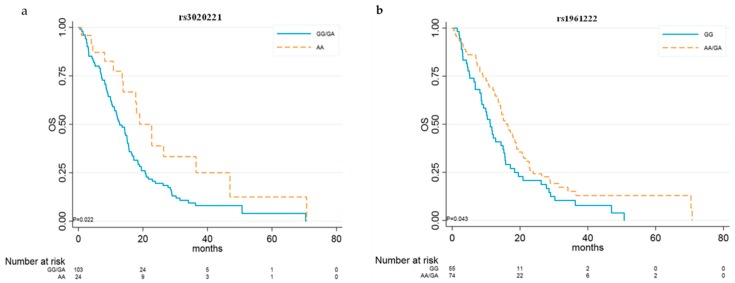
Kaplan–Meier curves for *ANGPT2* rs3020221 and rs1961222. OS in relation to rs3020221 genotypes (**a**) and rs1961222 genotypes (**b**).

*ANGPT2* rs3020221 and rs1961222 were associated only with OS. For rs3020221, we chose the recessive genetic model, and patients homozygous for the A allele showed significantly better median OS than did those with other genotypes (18.99 vs. 12.81 months, HR 0.53, 95% CI 0.31–0.92; *p* = 0.024) (Table 3 and Figure 2a). For rs1961222, we chose the dominant genetic model, and patients carrying at least one copy of the minor allele A showed significantly better median OS (16.43 vs. 11.24 months, HR 0.67, 95% CI 0.46–0.99; *p* = 0.044) than did those homozygous for the G allele (Table 3 and Figure 2b). No statistically significant differences were observed for other *ANGPT2* polymorphisms and PFS and OS.

**Figure 3:**
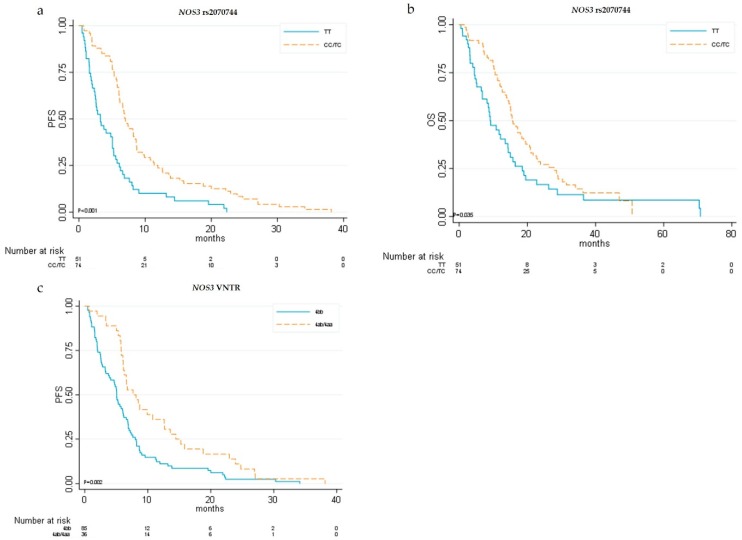
Kaplan–Meier curves for *eNOS* single-nucleotide polymorphisms (SNPs). (**a**) Progression-free survival (PFS) and (**b**) overall survival (OS) in relation to *eNOS* rs2070744T > C genotypes; (**c**) PFS in relation to *eNOS* VNTR4a/b genotypes.

With regard to *NOS3*, we conducted an updated follow-up of our previously described case series [6] and we added seven patients. By univariate analysis we confirmed that patients with at least one copy of the minor allele C for *NOS3* rs2070744T > C polymorphisms had a significantly better outcome, with higher median PFS (7.03 vs. 3.5 months, HR 0.43, 95% CI 0.30–0.63; *p* < 0.001) and OS (15.6 vs. 9.1 months, HR 0.65, 95% CI 0.44–0.97; *p =* 0.036) than those of patients homozygous for the T allele (Table 3 and Figure 3).

Moreover, *NOS3* VNTR4a/b patients with at least one copy of the minor allele “a” showed significantly higher median PFS (7.65 vs. 5.06 months, HR 0.54, 95% CI 0.36–0.80; *p* = 0.002) than did patients homozygous for the “b” allele (Table 3 and Figure 3).

No statistically significant differences were observed for the other *NOS3* polymorphisms. For *ANGPT2* SNPs, rs55633437 was associated with extra-hepatic spread; in particular, 64% of patients with at least one copy of the T allele presented with metastatic disease. Conversely, 32.7% of patients homozygous for the G allele showed extra-hepatic spread. No significant association was found between the main clinicopathological characteristics of patients and *NOS3* polymorphisms (analyses not shown).

Following adjustment for clinical covariates (age, etiology, and extra-hepatic spread), the final model of multivariate analysis confirmed *ANGPT2* rs55633437 and *NOS3* rs2070744 as the independent prognostic factors predicting PFS (HR 0.24, 95% CI 0.15–0.38, *p* < 0.001; HR 6.32, 95% CI 3.32–12.04, *p* < 0.001, respectively) and OS (HR 0.67, 95% CI 0.47–0.96, *p* = 0.03; HR 5.48, 95% CI 2.85–10.54, *p* < 0.001, respectively) (Table 4). Regarding the clinical parameters, extra-hepatic spread and HBV etiology remained the independent prognostic factors predicting OS (Table 4).

### 2.3. ANGPT2 and NOS3 Genotypes and Disease Control Rate (DCR)

Four (3.96%) patients showed a complete response (CR), 28 (27.72%) showed a partial response (PR), 34 (33.66%) showed stable disease (SD), and 35 (34.65) patients showed disease progression (PD). For 44 patients, information about the response was not available due to the retrospective nature of the study design. *ANGPT2* and *NOS3* polymorphisms were investigated in relation to the DCR. For *ANGPT2* polymorphisms, patients carrying at least one copy of the minor allele T for rs55633437 showed a lower percentage of DCR at the first CT re-evaluation than did those carrying the GG genotype (13.3% vs. 75.3%, respectively, *p* < 0.001). Patients carrying at least one copy of the minor allele A for rs1961222 showed a higher percentage of DCR at the first CT re-evaluation than did those carrying the GG genotype (75.4% vs. 48.8%, respectively; *p* = 0.007).

Regarding *NOS3*, patients carrying at least one copy of the minor allele C for *NOS3* rs2070744 showed a higher percentage of DCR at the first CT re-evaluation than did those carrying the TT genotype (81.1% vs. 48.8%, respectively; *p* = 0.001). No substantial differences were seen between other SNPs and response.

### 2.4. ANGPT2 and NOS3 Genotypes and Toxicities

We also investigated the relationship between *ANGPT2* and *NOS3* polymorphisms and the main toxicities (skin toxicity, asthenia, and diarrhea). We divided these toxicities into early (within a month of sorafenib treatment) and late (after a month of treatment).

We found that *ANGPT2* rs1961222 and rs17063434 were associated with late skin toxicity with grade ≥ 2 (Common Terminology Criteria for Adverse Events (CTCAE) 4.0) (*p* = 0.030 and *p* = 0.003, respectively) (Figure S1).

No significant associations were observed between other *ANGPT2* polymorphisms and skin toxicity, asthenia, and diarrhea (Figure S1).

We found also that *NOS3* rs1799983 was associated with late skin toxicity (*p* = 0.021) and with a higher grade (CTCAE 4.0) of this toxicity (*p* = 0.003).

### 2.5. Haplotypes Analysis and Clinical Outcomes

We observed linkage disequilibrium between *ANGPT2* polymorphisms. Lewontin’s disequilibrium coefficient (D’) and the correlation coefficient (r^2^) are reported in Figure S2.

We identified two blocks of SNPs using Haploview software version 4.2, and for both blocks we identified a total of four haplotypes. For Block 1, including rs1961222 and rs3020221, the most frequent haplotype was HT1 (G–G at rs302022/rs1961222) (57.1%), followed by HT4 (A–A) (38%), HT3 (A–G) (5.2%), and HT2 (G–A) (1%).

For Block 2, including rs3739392, rs3739391, and rs3739390, the most frequent haplotype was HT1 (T–C–G at rs3739392/rs3739391/rs3739390) (80.2%), followed by HT4 (C–T–C) (9.1%), HT3 (C–T–G) (6.3%), and HT2 (T–T–G) (4.4%).

Regarding Block 1, univariate analysis showed that patients carrying at least one copy of HT1 had lower median OS than did those without any copies of HT1 (12.8 vs. 21.7 months; HR 1.75, 95%CI 1.04–2.95; *p* = 0.037) (Table S3). No statistically significant differences were observed for other *ANGPT2* haplotypes of Block 1 in relation to PFS and OS (Table S3).

**Figure 4:**
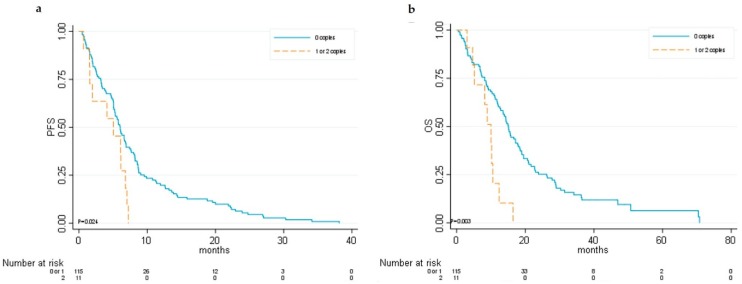
Kaplan–Meier curves for *ANGPT2* haplotype 2 (Block 2). (**a**) Progression-free survival (PFS) and (**b**) overall survival (OS).

Interestingly, regarding Block 2, univariate analysis showed that patients carrying at least one copy of HT2 had lower median PFS (5.03 vs. 6.04 months; HR 2.05, 95%CI 1.08–3.89; *p* = 0.027) and OS (9.9 vs. 15.1 months; HR 2.71, 95%CI 1.37–5.38; *p* = 0.004) than did those without any copies of HT2 (Table S4 and Figure 4).

The final multivariable model including age, etiology, and extra-hepatic spread confirmed the previously mentioned *NOS3* and *ANGPT2* polymorphisms and haplotype 2 (HT2) of Block 2 as independent prognostic factors predicting PFS (HR 0.24, 95% CI 0.15–0.38, *p* < 0.001; HR 6.03, 95% CI 3.1–11.6, *p* < 0.001; HR 2.48, 95% CI 1.2–5.2, *p* = 0.015, respectively) and OS (HR 0.46, 95% CI 0.29–0.73, *p* = 0.001; HR 4.88, 95% CI 2.99–11.5, *p* < 0.001; HR 2.30, 95% CI 1.02–5.2, *p* = 0.044, respectively). Regarding the clinical parameters, extra-hepatic spread and HBV etiology remained the independent prognostic factors predicting OS (Table S5).

### 2.6. Clustering Analysis

Unsupervised clustering of patients based on *ANGPT2* and *NOS3* non-synonymous variant profiles revealed two distinct groups: one exclusively characterized by variants in the regulatory region of *NOS3* (called “1”) and the other mainly affected by variations at the *ANGPT2* 5′ UTR (called “2”) (Figure S3).

Forty-four (32.5%) patients belonged to Cluster 1 and another 44 (32.5%) to Cluster 2. The median PFS was 6.1 months (95% CI 4.1–7.0) for patients of Cluster 2 and 7.4 months (95% CI 5.8–8.8) for patients of Cluster 1 (HR 1.55, 95% CI 0.99–2.41; *p* = 0.051) (Figure S4). The median OS was 10.6 months (95% CI 8.7–16.4) for patients of Cluster 2 and 17.2 months (95% CI 14.4–22.7) for patients of Cluster 1 (HR 1.74, 95% CI 1.08–2.80; *p* = 0.02) (Figure S4).

Following adjustment for clinical covariates (alpha-fetoprotein, extra-hepatic spread, and etiology), multivariate analysis confirmed that variants in the regulatory region of *NOS3* were independent prognostic factors predicting OS (HR 0.44, 95% CI 0.23–0.82; *p* = 0.01).

## 3. Discussion

The present study analyzed *ANGPT2* and *NOS3* polymorphisms in relation to clinical outcome in patients with advanced HCC receiving sorafenib. In particular, we found that patients with *ANGPT2* rs55633437 TT/GT genotypes had significantly lower median OS and PFS than did patients with other genotypes, and we confirmed that patients with the *NOS3* rs2070744 TT genotype showed a worse prognosis than did patients with other genotypes.

We also identified an *ANGPT2* haplotype (characterized by *ANGPT2* rs3739392, rs3739391, and rs3739390) that was significantly associated with worse OS and PFS.

Moreover, we found that patients exclusively characterized by variants in the regulatory region of *NOS3* showed better prognosis than did patients affected by variations at the *ANGPT2* 5′UTR.

We also found that patients with HCV etiology and without extra-hepatic spread showed better outcomes in terms of OS, in agreement with Bruix et al.’s pooled analysis [7,8]. They demonstrated that the benefit of sorafenib is significantly higher in patients with disease confined to the liver (without extra-hepatic spread), with HCV, or with low NLR (neutrophil/lymphocyte ratio).

We also found that patients carrying at least one copy of the minor allele T for *ANGPT2* rs55633437 and patients with *NOS3* rs2070744 TT genotypes showed a lower percentage of DCR at the first CT re-evaluation. Moreover, patients with other genotypes associated with better PFS and OS showed higher response rates.

*ANGPT2* rs55633437 and *NOS3* rs2070744 polymorphisms could identify a group of patients more resistant to sorafenib.

Currently, the measurement of specific predictive biomarkers for cancer therapy is mandatory in patients with various cancer types [9]. However, for HCC, no biomarkers are available in relation to sorafenib efficacy [3].

In the literature, only few studies have identified possible predictive markers relating to sorafenib in HCC patients. In the SHARP trial, Llovet and co-workers found that low VEGF-A and Ang-2 plasma baseline concentrations predicted survival in patients with advanced HCC and that high baseline plasma Ang-2 levels were related with a more aggressive disease [5]. Ang-2 protein levels also increased during treatment in their placebo group, whereas they remained constant during treatment with sorafenib, reflecting the generally more favorable outcome of patients in the sorafenib-treated group [5]. In agreement with Llovet’s study, Miyahara et al. reported that high baseline Ang-2 serum levels were associated with poor outcome in advanced-HCC patients receiving sorafenib [10].

Other authors investigated some SNPs in relation to sorafenib treatment. In particular, Scartozzi et al. in the ALICE-1 study [11] and Faloppi et al. in the ALICE-2 study [12] showed that SNPs in the *VEGF-A*, *VEGF-C*, and *HIF-1*α genes were independent factors influencing PFS and OS in HCC patients receiving sorafenib.

Our study demonstrated the role of *ANGPT2* and *NOS3* polymorphisms in relation to clinical outcome in advanced-HCC patients receiving sorafenib.

The *ANGPT2* gene is a highly polymorphic gene [13], and SNPs may alter gene expression [14]. Some SNPs have been studied in association with obstetric diseases, premature retinopathy, and acute respiratory distress syndrome [15,16,17].

Some authors investigated the role of *ANGPT2* variants in colorectal cancer patients with liver metastases [18] or in breast cancer patients [19] in relation to bevacizumab-based treatment, but no work has studied the impact of Ang-2 genetic variants in relation to treatment in HCC patients.

The functional role of our *ANGPT2* polymorphisms are not well documented in the literature, but SNP function prediction tools reveal that these SNPs could be located inside transcription factor binding sites (TFBS) or exonic splicing enhancers/silencers (ESE or ESS). In particular, the three SNPs of Block 2 (rs3739390, rs3739391, and rs3739392), located in the 5′ UTR region, could be found in a transcription factor binding site and probably have an effect on protein synthesis.

In our study, *ANGPT2* rs55633437 TT/GT genotypes and an *ANGPT2* haplotype were associated with lower median OS and PFS. Considering that increased Ang-2 expression levels were associated with poorer outcome in previous studies [5,10], it is plausible that these kinds of variants are associated with higher Ang2 protein levels.

Thus, it will be interesting to evaluate a correlation between the presence of a specific allele on a polymorphic site and the expression of the respective protein.

*ANGPT2* and *NOS3* are not the direct target of sorafenib, and other factors may be involved in the relation between Ang-2, NOS3 activity, and sorafenib efficacy. In particular, it is possible that these genetic variants are linked with other functional variants in the regulatory regions of the *ANGPT2* or *NOS3* genes.

With regard to toxicity, we found that *ANGPT2* rs1961222 and rs17063434 and *NOS3* rs1799983 were associated with late skin toxicity.

The development of dermatologic adverse events (DAEs) early (within the first 60 days of treatment) after treatment initiation is associated with delayed tumor progression and improved survival [20]. It was recently demonstrated that the angiotensinogen (AGT) M235T SNPs can predict early DAEs in HCC patients treated with sorafenib [21].

The identification of predictive biomarkers for early DAEs would be important to defining a population with a major survival impact by treatment.

It was shown that patients with hypertension during sorafenib treatment showed better PFS and OS [22,23,24]. Unfortunately, we did not have available data about hypertension due to the retrospective nature of the study, but given the possible correlation between *NOS3* polymorphisms and hypertension [25,26], it will be interesting to evaluate this in our ongoing prospective study [27].

The study has some limitations, e.g., its retrospective nature (cases were, however, consecutively selected, thus reducing potential bias). As a consequence, we were not able to collect detailed data on toxicities, particularly on hypertension and on the neutrophil/lymphocyte ratio (NLR).

Other limitations are that this study was carried out on Caucasian individuals only and the lack of a control arm without sorafenib. Thus, it is not possible to define the prognostic or predictive value of *ANGPT2* and *NOS3* polymorphisms.

## 4. Materials and Methods

### 4.1. Patient Enrollment

This was a retrospective multicenter Italian study carried out on 135 HCC patients consecutively treated at Istituto Scientifico Romagnolo per lo Studio e la Cura dei Tumori and at the Universities of Ancona, Milan, and Bari, from 2012 to 2015.

We considered patients with advanced or intermediate-stage HCC, diagnosed according to the American Association for the Study of Liver Diseases (AASLD) guidelines, treated with sorafenib, and refractory or no longer amenable to locoregional therapies. The eligibility criteria were the same as those of Llovet’s study [2]. Sorafenib was administered according to the agreed schedule (400 mg twice a day continuously), and dose modifications were applied when medically indicated. A CT/MRI scan every 8 weeks or as clinically indicated was used to provide follow-up monitoring. The modified Response Evaluation Criteria in Solid Tumors (mRECIST) was used to measure tumor response to treatment [28].

Treatment with sorafenib was continued until disease progression, unacceptable toxicity, or death. The study was approved by the Local Ethics Committees of each center and informed consent was obtained from each patient for their biological material to be used for research purposes (CEIIAV IRSTB051).

### 4.2. DNA Isolation and Genotyping

We performed *ANGPT2* genotyping using DNA extracted from whole-blood samples.

Peripheral blood samples were collected in EDTA tubes, and genomic DNA was extracted from 200 μL of whole blood using a QIAamp DNA Minikit (Qiagen SPA, Milan, Italy) in accordance with the manufacturer’s instructions. DNA quantity and quality were assessed by Nanodrop 1000 (Celbio, Milan, Italy).

Genotyping was performed for eight *ANGPT2* SNPs (rs3739390, rs3739391, rs3739391, rs55633437, rs3020221, rs1961222, rs2916747, rs17063434) by standard PCR and direct sequencing analysis on an ABI 3130 Genetic Analyzer (Applied Biosystems). Primer sequences and PCR conditions are reported in Table S6. PCRs were performed starting from 50 ng of genomic DNA.

We selected these polymorphisms through a review of the Single Nucleotide Polymorphism database (dbSNP) (http://www.ncbi.nlm.nih.gov/SNP) and of medical literature.

Three SNPs (rs3739390, rs3739391, and rs3739392), located in the 5′ UTR region, were found in a transcription factor binding site and could affect gene transcription. *ANGPT2* rs55633437, rs3020221, rs1961222, rs2916747, and rs17063434 are synonymous variants located inside exonic splicing enhancers/silencers and they could affect gene transcription and splicing.

Polymorphism selection was done by also considering a minor allele frequency (MAF) above 5% (with only the exception of rs17063434).

The selection of three *NOS3* polymorphisms (*NOS3* rs2070744; VNTR 4a/b and *NOS3* rs1799983) and genotyping analyses of these were described in our previous study [6].

The localizations and refSNP (rs) numbers of these polymorphisms are shown in Figure S5.

All analyses were centralized at Biosciences Laboratory, IRST IRCCS (Meldola, Italy), and laboratory personnel were blinded to patient status when performing genotyping.

### 4.3. Statistical Analysis

Data were summarized as median, minimum, and maximum values for continuous variables and as absolute frequencies and percentages for categorical variables. The association between SNPs and patients or clinical categorical variables was assessed by means of chi-squared test or Fisher’s exact test, when appropriate, and among patients and clinical continuous variables by means of Wilcoxon–Mann–Whitney testing.

The two main time-to-event end-points considered were progression-free survival (PFS), defined as the time since the beginning of the treatment with sorafenib until disease progression or death by any cause (whichever occurred first), and overall survival (OS), defined as the time since start of treatment with sorafenib until death by any cause. Patients not experiencing the event of interest were censored at the last follow-up available.

The disease control rate (DCR) was defined as the proportion of patients with a complete or partial response or with stable disease. Pearson’s chi-squared test or Fisher’s exact test was used to evaluate the association between SNPs and DCR or toxicity, when appropriate.

The Kaplan–Meier (KM) method and log-rank test were used to compare PFS and OS between groups of patients. The median follow-up was computed on censored observations only. The median PFS and OS values and corresponding 95% confidence intervals (CIs) are reported.

SNPs were prescreened prior to statistical analyses to determine the correct genetic model by analyzing the Kaplan–Meier curves, following the approach by Savas et al. [29]. When the number of patients with the minor allele homozygous genotype (*n* ≤ 10) was not sufficient, the dominant genetic model was considered.

Hazard ratios (HRs) were estimated by means of the Cox proportional hazards regression model. The proportional hazard assumption was assessed graphically and the test was based on Schoenfeld residuals. Hardy–Weinberg equilibrium, linkage disequilibrium, and haplotype analyses were performed using the Haploview v. 4.2 software package [30]. The software presents Lewontin’s disequilibrium coefficient (D’), defined as a nonrandom association of alleles at two or more loci. The D’ coefficient is equal to 1 only if there is a perfect linkage disequilibrium (LD). Haplotype blocks were found using the Haploview v. 4.2 software package using the algorithm by Gabriel et al. [31]. The association between haplotypes and PFS or OS was found by means of the weighted haplotype combination method proposed by French et al. [32] using a dominant model due to low frequencies.

To select the variables to include in the final Cox models, one for PFS and one for OS, we proceeded as follows: we considered those variables significantly associated at the 10% level by univariate analysis in addition to SNPs found to be significantly associated at the level of 10% by univariate analysis or after adjustment for clinical covariates. Moreover, correlation among variables, especially among SNPs, was taken into consideration during variable selection. SNP correlation was measured by estimating tetrachoric correlation coefficients.

All statistical analyses were performed using STATA 15.0 statistical software (StataCorp, College Station, TX, USA), version 3.4.1.

### 4.4. Clustering Analysis Method

We annotated variants using Variant Effect Predictor (VEP) [33]. We clustered patients according to the non-synonymous variant profiles (*ANGPT2* rs3739390, rs3739391, rs3739391, *NOS3* rs2070744; VNTR 4a/b) by assigning a numerical value of 1 or 2 if heterozygous or homozygous for a given variant and 0 otherwise. Based on these numerical profiles, we calculated the distance between each patient and clustered them using a full linkage hierarchical algorithm. The *pdist* and *linkage* functions of scipy (https://www.scipy.org/) and the matplotlib python libraries were used for clustering and drawing, respectively.

## 5. Conclusions

In conclusion, our results suggest that the *NOS3* rs2070744 and *ANGPT2* rs55633437 polymorphisms and the presence of an *ANGPT2* haplotype may be capable of identifying a subset of HCC patients who are more resistant to sorafenib in terms of OS, PFS, and DCR. These data will be confirmed in our ongoing multicenter prospective study (NCT02786342).

## Figures and Tables

**Table 1:** Patient characteristics.

Clinical and Pathologic Variables	No. (%)
**Median age at start of treatment (min–max)**	70 (25–88)
**Gender**	
Male	109 (80.7)
Female	26 (19.3)
**Etiology**	
Metabolic syndrome	18 (13.3)
Alcoholic	10 (7.4)
Viral—HBV	22 (16.3)
Viral—HCV	78 (57.8)
Cryptogenic	7 (5.2)
**BCLC stage**	
B	37 (27.4)
C	98 (72.6)
**Child–Pugh**	
A	125 (92.6)
B	10 (7.4)
**ECOG Performance Status**	
0	83 (61.5)
1–2	52 (38.5)
**Sorafenib dose reduction**	
No	59 (61.5)
Yes	37 (38.5)
Missing data	39
**Extra-hepatic spread**	
No	81 (64.3)
Yes	45 (35.7)
**Serum α-FP level**	
≤400	54 (40)
>400	35 (25.9)
Missing data	46

Abbreviations: BCLC, Barcelona Clinic Liver Cancer; ECOG, Eastern Cooperative Oncology Group; α-FP, alpha-fetoprotein; HBV, hepatitis B virus; HCV, hepatitis C virus.

**Table 2:** PFS and OS in relation to baseline patient characteristics.

Clinical and Pathologic Variables	PFS			OS		
	Median PFS [95% CI]	HR [95% CI]	*p*	Median OS [95% CI]	HR [95% CI]	*p*
**Gender**						
Female	6.11 [3.22–8.18]	1		12.35 [5.72–20.89]	1	
Male	5.62 [5.03–6.27]	0.96 [0.62–1.50]	0.873	14.85 [11.83–15.80]	0.94 [0.59–1.52]	0.814
Median age at start of treatment *		0.92 [0.85–0.995]	0.038		0.94 [0.87–1.02]	0.118
**Etiology**						
Viral—HCV	6.11 [5.06–6.90]	1		14.29 [11.14–17.77]	1	
Cryptogenic	3.98 [3.19–21.91]	0.96 [0.44–2.11]	0.926	16.43 [3.98–NR]	0.70 [0.29–1.76]	0.453
Alcoholic	5.26 [1.41–8.71]	0.98 [0.50–1.92]	0.956	14.39 [1.41–17.15]	1.23 [0.59–2.59]	0.580
Metabolic syndrome	6.01 [3.25–8.51]	1.21 [0.71–2.06]	0.478	15.64 [6.80–21.68]	1.11 [0.63–1.96]	0.710
Viral—HBV	5.06 [2.33–6.90]	1.45 [0.90–2.34]	0.131	8.57 [4.66–15.24]	1.95 [1.17–3.26]	0.011
**Child–Pugh**						
A	5.75 [5.06–6.60]	1		14.59 [11.83–15.74]	1	
B	6.11 [0.69–11.37]	0.76 [0.37–1.57]	0.459	13.53 [1.48–26.41]	1.16 [0.56–2.40]	0.683
**BCLC**						
B	6.60 [5.06–8.77]	1		14.36 [11.24–16.43]	1	
C	5.32 [4.11–6.14]	1.27 [0.86–1.88]	0.225	14.59 [9.99–16.43]	1.05 [0.68–1.62]	0.819
**ECOG Performance Status**						
0	5.75 [5.03–6.60]	1		14.39 [11.7–15.74]	1	
1–2	6.01 [2.69–7.42]	1.01 [0.71–1.45]	0.949	15.01 [8.18–18.92]	1.23 [0.84–1.80]	0.294
**Extra-hepatic spread**						
Yes	2.83 [1.94–5.22]	1		10.84 [6.96–15.08]	1	
No	6.27 [5.72–7.65]	0.50 [0.34–0.73]	<0.001	15.60 [12.81–18.00]	0.65 [0.43–0.99]	0.043
**Serum α-FP level**						
**≤400**	5.75 [3.75–6.64]	1		13.57 [10.35–16.66]	1	
**>400**	5.72 [2.92–7.23]	0.87 [0.56–1.34]	0.526	13.86 [8.15–15.80]	1.26 [0.80–2.01]	0.320

* 5-year increment. Abbreviations: BCLC, Barcelona Clinic Liver Cancer; ECOG, Eastern Cooperative Oncology Group; α-FP, alpha-fetoprotein; PFS, progression-free survival; OS, overall survival; HR, hazard ratio; HBV, hepatitis B virus; HCV, hepatitis C virus.

**Table 3:** Univariate analysis of PFS and OS in relation to *ANGPT2* and *NOS3* polymorphisms.

Gene Polymorphisms	Genetic Model	PFS			OS		
		Median PFS [95% CI]	HR [95% CI]	*p*	Median OS [95% CI]	HR [95% CI]	*p*
***ANGPT2***							
**rs3739392**	DOM						
TT		5.62 [5.03–6.73]	1		14.39 [11.24–16.43]	1	
CC/TC		6.14 [3.91–8.54]	0.94 [0.64–1.39]	0.765	13.57 [8.15–18.00]	0.92 [0.60–1.39]	0.679
**rs3739391**	DOM						
CC		5.75 [5.06–6.90]	1		15.11 [12.65–18.50]	1	
TT/CT		6.04 [3.91–6.80]	1.13 [0.79–1.63]	0.506	11.14 [8.54–15.64]	1.17 [0.79–1.73]	0.445
**rs3739390**	DOM						
GG		5.75 [5.03–6.60]	1		14.36 [11.24–15.74]	1	
CC/GC		6.27 [2.60–12.61]	0.83 [0.52–1.31]	0.416	12.81 [8.15–20.89]	0.88 [0.54–1.44]	0.621
**rs55633437**	DOM						
GG		6.27 [5.75–7.23]	1		15.51 [13.57–18.40]	1	
TT/GT		1.58 [0.76–3.32]	4.79 [2.73–8.35]	<0.001	4.66 [2.69–8.57]	4.86 [2.73–8.67]	<0.001
**rs3020221**	REC						
GG/GA		5.78 [5.06–6.60]	1		12.81 [10.35–15.24]	1	
AA		8.77 [4.01–10.78]	0.72 [0.45–1.14]	0.163	18.99 [13.57–36.47]	0.53 [0.31–0.92]	0.024
**rs1961222**	DOM						
GG		5.32 [3.19–6.60]	1		11.24 [8.54–15.01]	1	
AA /GA		6.04 [5.09–8.02]	0.93 [0.65–1.34]	0.712	16.43 [13.57–18.99]	0.67 [0.46–0.99]	0.044
**rs17063434**	DOM						
TT		5.62 [5.03–6.14]	1		0.84 [11.99–17.77]	1	
CC /TC		6.80 [6.14–8.54]	0.93 [0.55–1.56]	0.779	11.24 [8.15–15.64]	1.59 [0.94–2.69]	0.084
**rs2916747**							
TT		5.78 [5.06–6.60]	1		14.39 [11.99–16.43]	1	
TC		6.27 [1.97–13.83]	0.61 [0.34–1.10]	0.102	11.70 [4.20–27.79]	1.09 [0.61–1.94]	0.784
***NOS3***							
***NOS3* +894 (rs1799983)**	DOM						
GG		5.22 [2.83–6.11]	1		15.74 [9.23–18.50]	1	
GT/TT		6.60 [5.32–8.18]	0.81 [0.57–1.16]	0.251	14.29 [11.14–15.51]	1.14 [0.77–1.68]	0.527
**VNTR4a4b**	DOM						
4bb		5.06 [3.75–6.11]	1		11.99 [9.10–14.85]	1	
4aa/4ab		7.65 [6.08–12.61]	0.54 [0.36–0.80]	0.002	17.15 [14.59–20.89]	0.68 [0.44–1.05]	0.080
***NOS3*-786 (rs2070744)**	DOM						
TT		3.25 [2.33–5.06]	1		9.10 [6.80–14.29]	1	
CC/TC		7.03 [6.08–8.67]	0.43 [0.30–0.63]	<0.001	15.60 [13.86–19.51]	0.65 [0.44–0.97]	0.036

Abbreviations: DOM, dominant; REC; recessive; VNTR, variable number of tandem repeats; PFS, progression-free survival; OS, overall survival; HR, hazard ratio.

**Table 4:** Multivariate analysis of OS.

Patient Characteristics	HR [95% CI]	*p*
**Extra-hepatic spread**		
Yes	1	
No	0.54 [0.35–0.84]	0.007
**Etiology**		
Viral—HCV	1	
Biliary cirrhosis/cryptogenic	0.33 [0.08–1.39]	0.131
Alcoholic	1.69 [0.75–3.84	0.207
Metabolic syndrome	1.50 [0.80–2.83]	0.209
Viral—HBV	2.42 [1.38–4.26]	0.002
***NOS3*-786 (rs2070744)**		
TT	1	
CC/TC	0.67 [0.47–0.96]	0.030
***ANGPT2* rs55633437**		
GG	1	
TT/GT	5.48 [2.85–10.54]	<0.001

## Supplementary Materials

The following are available online at https://www.mdpi.com/2072-6694/11/7/1023/s1, Figure S1: Relationship between *ANGPT2* polymorphisms and the main toxicities, Figure S2: Haploview linkage disequilibrium plot and identification of haplotype block in the *ANGPT2* gene, Figure S3: Clustering of non-synonymous variants, Figure S4: Kaplan–Meier curves in accordance with non-synonymous variant clusters, Figure S5: *ANGPT2* and *NOS3* polymorphisms, Table S1: Genotype frequencies of *ANGPT2* and *NOS3* polymorphisms, Table S2: Correlation coefficient between polymorphisms, Table S3: Univariate analysis of PFS and OS in relation to Block 1 *ANGPT2* haplotypes, Table S4: Univariate analysis of PFS and OS in relation to Block 2 *ANGPT2* haplotypes, Table S5: Multivariate analysis of OS, considering *NOS3*, *ANGPT2* SNPs and haplotypes, Table S6: Primer sequences for *ANGPT2* SNPs and PCR programs.

## References

[1] The Global Cancer Observatory Globocan 2018. Http://globocan.Iarc.Fr/.

[2] Llovet J.M., Ricci S., Mazzaferro V., Hilgard P., Gane E., Blanc J.F., de Oliveira A.C., Santoro A., Raoul J.L., Forner A. (2008). Sorafenib in Advanced Hepatocellular Carcinoma. N. Engl. J. Med..

[3] Marisi G., Cucchetti A., Ulivi P., Canale M., Cabibbo G., Solaini L., Foschi F.G., De Matteis S., Ercolani G., Valgiusti M. (2018). Ten Years of Sorafenib in Hepatocellular Carcinoma: Are there any Predictive and/or Prognostic Markers?. World J. Gastroenterol..

[4] Kudo M., Finn R.S., Qin S., Han K.H., Ikeda K., Piscaglia F., Baron A., Park J.W., Han G., Jassem J. (2018). Lenvatinib Versus Sorafenib in First-Line Treatment of Patients with Unresectable Hepatocellular Carcinoma: A Randomised Phase 3 Non-Inferiority Trial. Lancet.

[5] Llovet J.M., Pena C.E., Lathia C.D., Shan M., Meinhardt G., Bruix J., SHARP Investigators Study Group (2012). Plasma Biomarkers as Predictors of Outcome in Patients with Advanced Hepatocellular Carcinoma. Clin. Cancer Res..

[6] Casadei Gardini A., Marisi G., Faloppi L., Scarpi E., Foschi F.G., Iavarone M., Lauletta G., Corbelli J., Valgiusti M., Facchetti F. (2016). ENOS Polymorphisms and Clinical Outcome in Advanced HCC Patients Receiving Sorafenib: Final Results of the ePHAS Study. Oncotarget.

[7] Bruix J., Cheng A.L., Meinhardt G., Nakajima K., De Sanctis Y., Llovet J. (2017). Prognostic Factors and Predictors of Sorafenib Benefit in Patients with Hepatocellular Carcinoma: Analysis of Two Phase III Studies. J. Hepatol..

[8] Casadei Gardini A., Frassineti G.L., Foschi F.G., Ercolani G., Ulivi P. (2017). Sorafenib and Regorafenib in HBV- Or HCV-Positive Hepatocellular Carcinoma Patients: Analysis of RESORCE and SHARP Trials. Dig. Liver Dis..

[9] Duffy M. (2017). Clinical use of Tumor Biomarkers: An Overview. Klin. Biochem. Metab..

[10] Miyahara K., Nouso K., Tomoda T., Kobayashi S., Hagihara H., Kuwaki K., Toshimori J., Onishi H., Ikeda F., Miyake Y. (2011). Predicting the Treatment Effect of Sorafenib using Serum Angiogenesis Markers in Patients with Hepatocellular Carcinoma. J. Gastroenterol. Hepatol..

[11] Scartozzi M., Faloppi L., Svegliati Baroni G., Loretelli C., Piscaglia F., Iavarone M., Toniutto P., Fava G., De Minicis S., Mandolesi A. (2014). VEGF and VEGFR Genotyping in the Prediction of Clinical Outcome for HCC Patients Receiving Sorafenib: The ALICE-1 Study. Int. J. Cancer.

[12] Faloppi L., Casadei Gardini A., Masi G., Silvestris N., Loretelli C., Ulivi P., Vivaldi C., Bianconi M., Giampieri R., Bittoni A. (2016). Angiogenesis Polymorphisms Profile in the Prediction of Clinical Outcome of Advanced HCC Patients Receiving Sorafenib: Combined Analysis of VEGF and HIF-1α. Final Results of the ALICE-2 Study. JCO.

[13] Ward E.G., Grosios K., Markham A.F., Jones P.F. (2001). Genomic Structures of the Human Angiopoietins show Polymorphism in Angiopoietin-2. Cytogenet. Cell Genet..

[14] Hegen A., Koidl S., Weindel K., Marme D., Augustin H.G., Fiedler U. (2004). Expression of Angiopoietin-2 in Endothelial Cells is Controlled by Positive and Negative Regulatory Promoter Elements. Arterioscler. Thromb. Vasc. Biol..

[15] Huber A., Grimm C., Pietrowski D., Zeillinger R., Bettendorf H., Husslein P., Hefler L. (2005). An Angiopoietin-2 Gene Polymorphism in Unexplained Intrauterine Fetal Death: A Multi-Center Study. J. Reprod. Immunol..

[16] Banyasz I., Bokodi G., Vannay A., Szebeni B., Treszl A., Vasarhelyi B., Tulassay T., Szabo A. (2006). Genetic Polymorphisms of Vascular Endothelial Growth Factor and Angiopoietin 2 in Retinopathy of Prematurity. Curr. Eye Res..

[17] Su L., Zhai R., Sheu C.C., Gallagher D.C., Gong M.N., Tejera P., Thompson B.T., Christiani D.C. (2009). Genetic Variants in the Angiopoietin-2 Gene are Associated with Increased Risk of ARDS. Intensive Care Med..

[18] Stremitzer S., Zhang W., Yang D., Ning Y., Stintzing S., Sebio A., Sunakawa Y., Yamauchi S., Matsusaka S., El-Khoueiry R. (2015). Genetic Variations in Angiopoietin and Pericyte Pathways and Clinical Outcome in Patients with Resected Colorectal Liver Metastases. Cancer.

[19] Makhoul I., Todorova V.K., Siegel E.R., Erickson S.W., Dhakal I., Raj V.R., Lee J.Y., Orloff M.S., Griffin R.J., Henry-Tillman R.S. (2017). Germline Genetic Variants in TEK, ANGPT1, ANGPT2, MMP9, FGF2 and VEGFA are Associated with Pathologic Complete Response to Bevacizumab in Breast Cancer Patients. PLoS ONE.

[20] Branco F., Alencar R.S., Volt F., Sartori G., Dode A., Kikuchi L., Tani C.M., Chagas A.L., Pfiffer T., Hoff P. (2017). The Impact of Early Dermatologic Events in the Survival of Patients with Hepatocellular Carcinoma Treated with Sorafenib. Ann. Hepatol..

[21] M.Reig M., Boix L., Torres F., Darnell A., DiazGonzalez A., Llarch N., Belmonte E., Sapena V., Zamparelli M.S., Corominas J. (2018). Towards Personalised Approach in Systemic Treatment for Hepatocellular Carcinoma. the Value of AGT M235T Gene Polymorphism. J. Hepatol..

[22] Estfan B., Byrne M., Kim R. (2013). Sorafenib in Advanced Hepatocellular Carcinoma: Hypertension as a Potential Surrogate Marker for Efficacy. Am. J. Clin. Oncol..

[23] Akutsu N., Sasaki S., Takagi H., Motoya M., Shitani M., Igarashi M., Hirayama D., Wakasugi H., Yamamoto H., Kaneto H. (2015). Development of Hypertension within 2 Weeks of Initiation of Sorafenib for Advanced Hepatocellular Carcinoma is a Predictor of Efficacy. Int. J. Clin. Oncol..

[24] Casadei Gardini A., Scarpi E., Marisi G., Foschi F.G., Donati G., Giampalma E., Faloppi L., Scartozzi M., Silvestris N., Bisulli M. (2016). Early Onset of Hypertension and Serum Electrolyte Changes as Potential Predictive Factors of Activity in Advanced HCC Patients Treated with Sorafenib: Results from a Retrospective Analysis of the HCC-AVR Group. Oncotarget.

[25] Merkus D., Sorop O., Houweling B., Boomsma F., van den Meiracker A.H., Duncker D.J. (2006). NO and Prostanoids Blunt Endothelin-Mediated Coronary Vasoconstrictor Influence in Exercising Swine. Am. J. Physiol. Heart Circ. Physiol..

[26] Wiley K.E., Davenport A.P. (2001). Physiological Antagonism of Endothelin-1 in Human Conductance and Resistance Coronary Artery. Br. J. Pharmacol..

[27] Casadei Gardini A., Faloppi L., Aprile G., Brunetti O., Caparello C., Corbelli J., Chessa L., Bruno D., Ercolani G., Leonetti A. (2017). Multicenter Prospective Study of Angiogenesis Polymorphism Validation in HCC Patients Treated with Sorafenib. an INNOVATE Study Protocol. Tumori.

[28] Lencioni R., Llovet J.M. (2010). Modified RECIST (mRECIST) Assessment for Hepatocellular Carcinoma. Semin. Liver Dis..

[29] Savas S., Liu G., Xu W. (2013). Special Considerations in Prognostic Research in Cancer Involving Genetic Polymorphisms. BMC Med..

[30] Barrett J.C., Fry B., Maller J., Daly M.J. (2005). Haploview: Analysis and Visualization of LD and Haplotype Maps. Bioinformatics.

[31] Gabriel S.B., Schaffner S.F., Nguyen H., Moore J.M., Roy J., Blumenstiel B., Higgins J., DeFelice M., Lochner A., Faggart M. (2002). The Structure of Haplotype Blocks in the Human Genome. Science.

[32] French B., Lumley T., Cappola T.P., Mitra N. (2012). Non-Iterative, Regression-Based Estimation of Haplotype Associations with Censored Survival Outcomes. Stat. Appl. Genet. Mol. Biol..

[33] McLaren W., Gil L., Hunt S.E., Riat H.S., Ritchie G.R., Thormann A., Flicek P., Cunningham F. (2016). The Ensembl Variant Effect Predictor. Genome Biol..

