# Supplementary material for: ANGPT2 and NOS3 Polymorphisms and Clinical Outcome in Advanced Hepatocellular Carcinoma Patients Receiving Sorafenib
Source: Cancers (Basel). 2019 Jul 20;11(7):1023. doi: 10.3390/cancers11071023 (PMC6679015; doi:10.3390/cancers11071023)
Supplement: Supplementary file 1 [file cancers-11-01023-s001.pdf]

Supplementary Materials: *ANGPT2* and *NOS3* Polymorphisms and Clinical Outcome in Advanced Hepatocellular Carcinoma Patients Receiving Sorafenib

Giorgia Marisi, Elisabetta Petracci, Francesco Raimondi, Luca Faloppi, Francesco Giuseppe Foschi, Gianfranco Lauletta, Massimo Iavarone, Matteo Canale, Martina Valgiusti, Luca Maria Neri, Paola Ulivi, Giulia Orsi, Giulia Rovesti, Ranka Vukotic, Fabio Conti, Alessandro Cucchetti, Giorgio Ercolani, Kalliopi Andrikou, Stefano Cascinu, Mario Scartozzi and Andrea Casadei-Gardini

Table S1. Genotype frequencies of *ANGPT2* and *NOS3* polymorphisms.

Gene Polymorphisms	No.	(%)
ANGPT2		
**rs3739392**		
CC	3	(2.33)
TC	34	(26.36)
TT	92	(71.32)
missing	6	
**rs3739391**		
CC	81	(62.79)
CT	45	(34.88)
TT	3	(2.33)
missing	6	
**rs3739390**		
CC	1	(0.78)
GC	22	(17.05)
GG	106	(82.17)
missing	6	
**rs55633437**		
GG	111	(87.40)
GT	14	(11.02)
TT	2	(1.57)
missing	8	
**rs3020221**		
AA	24	(18.90)
GA	58	(45.67)
GG	45	(35.43)
missing	8	
**rs1961222**		
AA	21	(16.28)
GA	53	(41.09)
GG	55	(42.64)
missing	6	
**rs17063434**		
CC	1	(0.78)
TC	18	(14.06)
TT	109	(85.16)
missing	7	
**rs2916747**		
TC	14	(10.94)
TT	114	(89.06)
missing	7	
NOS3		
***NOS3*+894 (rs1799983)**		
GG	58	(46.40)
GT	56	(44.80)
TT	11	(8.80)
missing	10	
**VTNR 4a4b**		
4aa	3	(2.48)
4ab	33	(27.27)

4bb	85	(70.25)
missing	14	
***NOS3*-786 (rs2070744)**		
CC	20	(16.00)
TC	54	(43.20)
TT	51	(40.80)
missing	10	

Abbreviations: VNTR, variable number of tandem repeats

Table S2. Correlation coefficient between polymorphisms.

Gene Polymorphisms	rs1799983 (DOM)	vntr4a4b (DOM)	rs2070744 (DOM)	rs3739392 (DOM)	rs3739391 (DOM)	rs3739390 (DOM)	rs55633437 (DOM)	rs3020221 (REC)	rs1961222 (DOM)	rs17063434 (DOM)	rs291674701
rs1799983 (DOM)	1.00										
vntr4a4b (DOM)	−0.26	1.00									
rs2070744 (DOM)	0.59 *	0.77 *	1.00								
rs3739392 (DOM)	−0.23	0.06	0.02	1.00							
rs3739391 (DOM)	−0.20	−0.02	0.06	1.00 *	1.00						
rs3739390 (DOM)	−0.26	0.09	−0.14	1.00 *	1.00*	1.00					
rs55633437 (DOM)	0.02	−0.20	0.17	0.17	0.30	−0.09	1.00				
rs3020221 (REC)	0.09	−0.08	−0.06	−0.06	−0.13	−0.02	−1.00	1.00			
rs1961222 (DOM)	−0.01	−0.13	0.03	0.11	0.08	0.06	−0.59 *	0.72 *	1.00		
rs17063434 (DOM)	0.07	0.12	0.01	0.14	0.16	0.31	−0.06	−1.00 *	−0.35	1.00	
rs291674701	0.46 *	0.10	0.18	0.24	0.11	0.01	0.19	−0.27	0.39	−0.02	1.00

* Values with an asterisk means that they differ statistically significantly from 0 (no correlation). Abbreviations: DOM, dominant; REC; recessive.

Table S3. Univariate analysis of PFS and OS in relation to Block 1 *ANGPT2* haplotypes.

***ANGPT2* Haplotypes**	**No. (%)**	**PFS**			**OS**		
		**Median PFS [95% CI]**	**HR [95% CI]**	***p***	**Median OS [95% CI]**	**HR [95% CI]**	***p***
**HT1 (G-G)**							
0 copies	26 (20.6)	8.21[5.03–11.37]	1		21.7 [13.9–NA]	1	
1 or 2 copies	100 (79.4)	5.78 [5.06–6.64]	1.33 [0.85–2.09]	0.213	12.8[10.6–15.5]	1.75 [1.04–2.95]	**0.037**
**HT2 (G-A)**							
0 copies	124 (98.7)	6.01 [5.22–6.8]	1		14.4[11.9–16.7]	1	
1 or 2 copies	2 (1.3)	5.27 [2.33–NA]	1.41 [0.35–5.74]	0.631	12.4 [3.19–NA]	1.40 [0.35–5.72]	0.64
**HT3 (A-G)**							
0 copies	114 (90.5)	6.01 [5.09–6.8]	1		14.3 [12.0–16.7]	1	
1 or 2 copies	12 (9.5)	6.07 [2.69–NA]	0.65 [0.35–1.23]	0.185	15.0 [10.8–NA]	0.76 [0.39–1.48]	0.42
**HT4 (A-A)**							
0 copies	52 (41.3)	5.32 [3.75–6.87]	1		11.2 [8.74–15.5]	1	
1 or 2 copies	74 (58.7)	6.04 [5.22–8.15]	0.97 [0.67–1.4]	0.862	16.4 [13.86–19.5]	0.68 [0.46–1.00]	0.05

Table S4. Univariate analysis of PFS and OS in relation to Block 2 *ANGPT2* haplotypes.

ANGPT2 Haplotypes	No. (%)	PFS			OS		
		Median PFS [95% CI]	HR [95% CI]	*p*	Median OS [95% CI]	HR [95% CI]	*p*
**HT1 (T-C-G)**							
0 copies	3 (2.4)	4.73 [1.02–NA]	1		7.39 [1.02–NA]	1	
1 or 2 copies	123 (97.6)	6.04 [5.22–6.87]	1.06 [0.34–3.36]	0.921	14.39 [11.9–17.1]	1.20 [0.36–4.05]	0.763
**HT2 (T-T-G)**							
0 copies	115 (91.3)	6.04 [5.26–6.9]	1		15.08 [12.81–18.4]	1	
1 or 2 copies	11 (8.7)	5.03 [1.97–NA]	2.05 [1.08–3.89]	**0.027**	9.99 [5.16–NA]	2.71 [1.37–5.38]	**0.004**
**HT3 (C-T-G)**							
0 copies	111 (88.1)	6.04 [5.22–6.90]	1		14.6 [11.9–17.1]	1	
1 or 2 copies	15 (11.9)	6.01 [2.50–9.89]	1.33 [0.77–2.3]	0.303	13.6 [7.39–NA]	1.13 [0.62–2.07]	0.689
**HT4 (C-T-C)**							
0 copies	104 (82.5)	5.75 [5.06–6.8]	1		14.4 [11.83–16.7]	1	
1 or 2 copies	22 (17.5)	6.45 [3.22–13.8]	0.82 [0.52–1.31]	0.407	14.2 [8.74–28.9]	0.86 [0.52–1.40]	0.536

Table S5. Multivariate analysis of OS, considering *NOS3*, *ANGPT2* SNPs and haplotypes.

Patient Characteristics	HR [95% CI]	*p*
**Extrahepatic spread**		
Yes	1	
No	0.56 [0.36–0.89]	**0.015**
**Etiology**		
Viral-HCV	1	
Biliary cirrhosis/cryptogenic	0.28 [0.07–1.2]	0.088
Alcoholic	2.04 [0.89–4.68]	0.092
Metabolic syndrome	1.58 [0.84–2.99]	0.158
Viral-HBV	2.57 [1.43–4.59]	**0.002**
***ANGPT2* rs55633437**		
GG	1	
TT/GT	4.88 [2.99–11.53]	**<0.001**
***NOS3*-786 (rs2070744)**		
TT	1	
CC/TC	0.46 [0.29–0.73]	**0.001**
***ANGPT2* HT2 (T-T-G)**		
0 copies	1	
1 or 2 copies	2.30 [1.02–5.19]	**0.044**

Table S6. Primer sequences for *ANGPT2* SNPs and PCR programs.

SNPs	Primer Sequences (5′-3′)	PCR Programs
ANGPT2		
rs3739390<br>rs3739391<br>rs3739392	F: CCTGGAGAGAACACAGCAGT<br>R: CGGCCAAGACAAGATCACAG	Step 1: 39 cycles of 94 °C for 60 s, 62 °C for 60 s and 72 °C) for 60 s; Step2: 72 °C for 5 min
rs3020221<br>rs55633437	F: GCTACAGGTGTTAGTATCCAAGC<br>R: TGAGAAATAGCGCCTTTTCTGA	Step 1: 39 cycles of 94 °C for 60s, 58 °C for 60 s and 72 °C for 60 s; Step2: 72 °C for 5 min
rs1961222	F: AGGACCCCACTGTTGCTAAA<br>R: GTGAGGCTGGGGAAGATCTT	Step 1: 39 cycles of 94°C for 60 s, 62°C for 60 s and 72 °C for 60 s; Step2: 72 °C for 5 min
rs17063434<br>rs2916747	F: ACTTGCATTACAGGGATTTGGT<br>R: GCCCGGCCACAAATCTTTTA	Step 1: 39 cycles of 94 °C for 30s, 60 °C for 30 s and 72 °C for 30 s; Step2: 72 °C for 5 min

Abbreviations: F, forward primer; R, reverse primer.

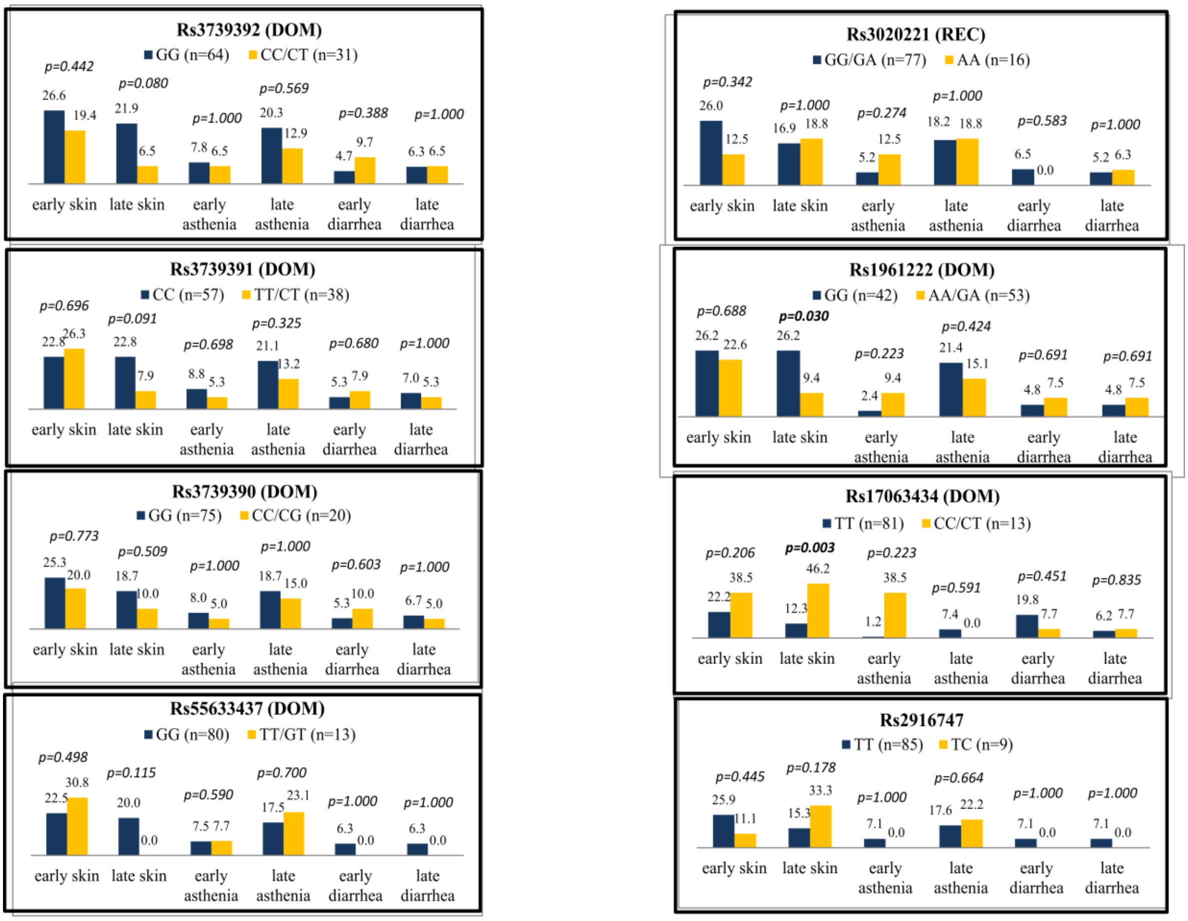

Figure S1. Relationship between *ANGPT2* polymorphisms and the main toxicities.

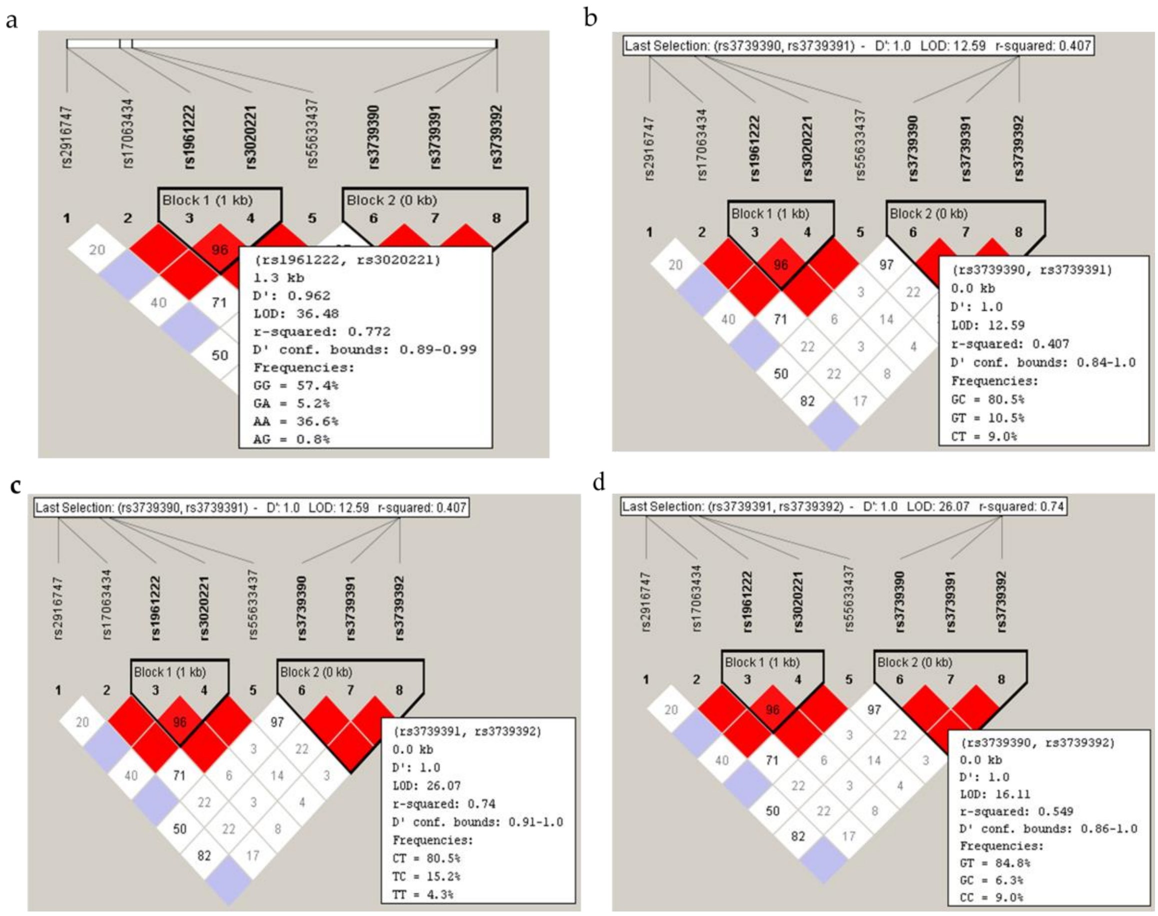

Figure S2. Haploview linkage disequilibrium plot and identification of haplotype block in *ANGPT2* gene. Lewontin's disequilibrium coefficient (D') and correlation coefficient (r^2) between the two SNPs of Block 1 (**a**); rs3739390 and rs3739391of Block 2 (**b**); rs3739391 and rs3739392of Block 2 (**c**); rs3739390 and rs3739392of Block 2 (**d**). Pairwise linkage disequilibrium (LD) coefficients D'×100, indicating extent of LD between SNPs, are shown in each square (D' values of 1.0 are not shown). Higher color intensity of the squares indicates higher LD between SNPs. The inverted black triangle represents a single haplotype block.

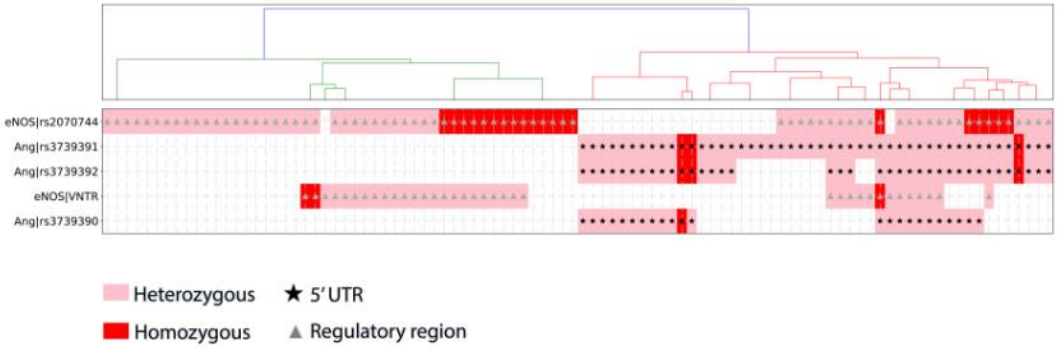

Figure S3. Clustering of non-synonymous variants. Green cluster was exclusively characterized by variants in the regulatory region of *NOS3* (gray triangles), the red cluster was mainly affected by variations at the *ANGPT2* 5' UTR (marked by black stars).

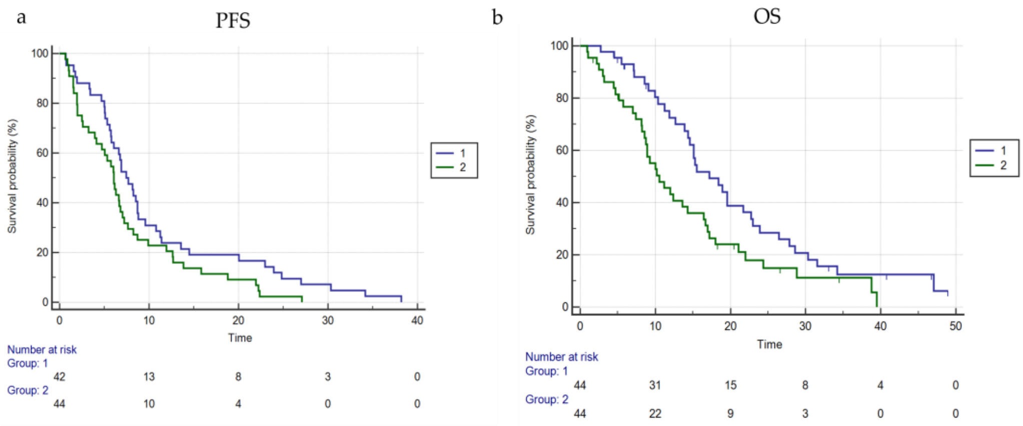

Figure S4. Kaplan Meier curves in accordance with non-synonymous variant clusters. (**A**) Progression-free survival (PFS) and (**B**) overall survival (OS). Cluster "1" represent the cluster.

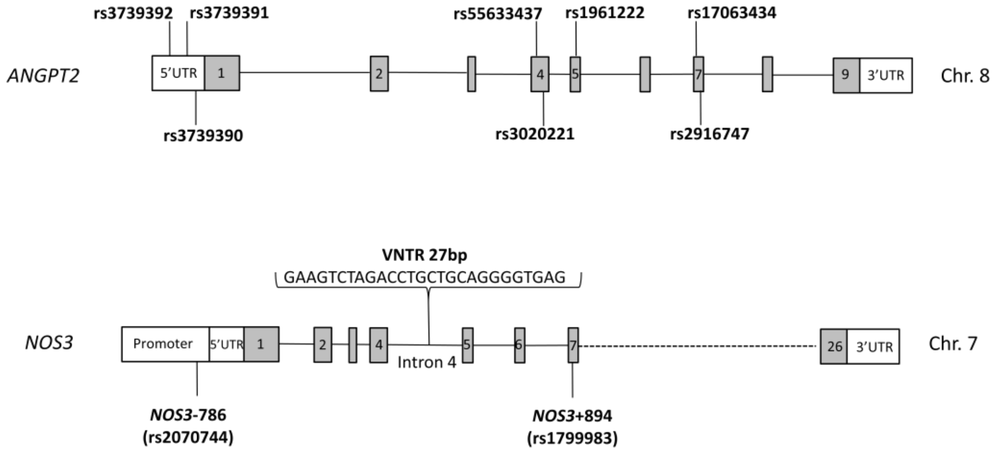

Figure S5. *ANGPT2* and *NOS3* polymorphisms. Localization and rs reference numbers of the polymorphisms analyzed in the study. Gray rectangles represent the exons, while the lines are introns of the gene.

© 2019 by the authors. Licensee MDPI, Basel, Switzerland. This article is an open access article distributed under the terms and conditions of the Creative Commons Attribution (CC BY) license (http://creativecommons.org/licenses/by/4.0/).
